# Prevalence of Serum Antibody Titers against Core Vaccine Antigens in Italian Cats

**DOI:** 10.3390/life13122249

**Published:** 2023-11-23

**Authors:** Paola Dall’Ara, Stefania Lauzi, Lauretta Turin, Francesco Servida, Laura Barbieri, Jari Zambarbieri, Giulia Mazzotti, Federico Granatiero, Elena Scarparo, Aurora Mirabile, Stefano Bo, Joel Filipe

**Affiliations:** 1 Department of Veterinary Medicine and Animal Sciences (DIVAS), University of Milan, Via dell’Università 6, 26900 Lodi, LO, Italyjoel.soares@unimi.it (J.F.); 2 Clinica Veterinaria Pegaso, Via Dante Alighieri 169, 22070 Rovello Porro, CO, Italy; 3 Clinica Veterinaria Turro, Via Gerolamo Rovetta 8, 20127 Milano, MI, Italy; 4 Ambulatorio Veterinario Mazzotti, Via Papa Giovanni XXIII 93, 24054 Calcio, BG, Italy; 5 Clinica Veterinaria Prealpi, Via Monviso, 22070 Limido Comasco, CO, Italy; 6 Ambulatorio Veterinario Bo-Ferro, Via Fratelli Calandra 3, 10123 Torino, TO, Italy

**Keywords:** cat, core vaccinations, Feline panleukopenia virus (FPV), Felid herpesvirus type 1 (FeHV-1), Feline calicivirus (FCV), antibody titration, VacciCheck

## Abstract

Feline core vaccines strongly recommended for all cats are against Feline panleukopenia virus (FPV), Felid herpesvirus type 1 (FeHV-1), and Feline calicivirus (FCV), but cats can be classified as low- and high-risk based on their lifestyle. The aim of this study was to determine the actual seroprotection against FPV, FeHV-1, and FCV in a large cohort of Italian cats by using the VacciCheck test. A total of 740 cats (567 owned and 173 stray cats; 435 vaccinated and 305 unvaccinated) were analyzed for Protective Antibody Titers (PATs). Differences related to origin, sex, age, breed, FIV/FeLV status, health status, and time elapsed since last vaccination were evaluated. Less than half of the entire cohort (36.4%) had PATs for all three diseases simultaneously, increasing to 48.6% if weak positive values were also considered and 50.3% when considering only the 435 vaccinated cats. Particularly, antibodies were detected against FCV, FPV, and FeHV-1 at protective titers (PATs) in 78.6%, 68.1, and 49.1% of the cats, respectively. In general, owned, neutered, and adult FIV- and/or FeLV-negative cats were the most protected categories, even if not always for the three viruses. Most cats maintained high PATs for 3 years or longer after vaccination against FPV and FCV but not FeHV-1. Long-lasting protective immunity persisted for many years after the last vaccination (more than 18 years in the oldest cats). Nevertheless, since not all cats were protected after so many years and for all pathogens, checking protection via antibody titration could be the best choice to prevent immunity breakdowns. The discussion also focuses on the reliability of antibody titration for the two URTD (upper respiratory tract disease) viruses which, unlike for FPV, is not widely accepted as a valid index of protection.

## 1. Introduction

Several authors repeatedly mentioned that vaccination is the most important and successful tool for the prevention of widespread, life-threatening diseases [1]. When looking at the worldwide situation for cats, some associations recently published vaccination guidelines specifically devoted to them, such as the American Animal Hospital Association (AAHA), together with the American Association of Feline Practitioners (AAFP) [2,3] and the European Advisory Board on Cat Diseases (ABCD) [4,5], while others have drawn up guidelines that apply to both cats and dogs (i.e., the World Small Animal Veterinary Association (WSAVA), which produced global [6], Asian [7], and Latino-American [8] versions, the Australian Veterinary Association (AVA) [9], the British Veterinary Association (BVA) [10], and the Canadian Veterinary Medical Association (CVMA) [11,12]). All these guidelines classify cat and dog vaccines as core or non-core. Core vaccines are intended for all pets around the world since they protect against dangerous, contagious, and widely spread diseases, while non-core ones are optional and recommended only for animals really at risk of contracting some diseases.

Feline panleukopenia virus (FPV) is a small, non-enveloped DNA virus of the family *Parvoviridae*, subfamily *Parvovirinae*, genus *Parvovirus*, and species Carnivore protoparvovirus 1 that is closely related to Canine parvovirus type 2 (CPV-2), which is believed to be derived from the former [13]. Cats can also be infected by new CPV-2 strains (2a, 2b, and 2c) and become sick [14,15]. FPV is responsible for a widespread, highly contagious, and lethal disease, with epidemics affecting mainly kittens (3–5 months old), especially in the summer–autumn period, but adult cats are not spared from infection if not correctly vaccinated. Transmission can occur via both direct (fecal–oral route) and indirect contact due to the long resistance and persistence of the virus in the environment. The disease causes predominantly gastroenteric and neurological clinical signs (especially cerebellar hypoplasia in kittens), as well as marked leukopenia accompanied by immunosuppression [13,16,17,18,19].

Felid herpesvirus type 1 (FeHV-1) is an enveloped (and therefore labile in the environment) DNA virus of the family *Herpesviridae*, subfamily *Alphaherpesvirinae*, and genus *Varicellovirus*. It is responsible for feline viral rhinotracheitis and, together with Feline calicivirus (FCV, see later) and other minor pathogens (i.e., *Chlamydophila felis*, *Bordetella bronchiseptica*), it is involved in the etiopathogenesis of upper respiratory tract disease (URTD). This virus typically affects kittens and juvenile cats when maternal passive immunity wanes. As for the other herpesviruses, acute infections are followed by lifelong latency in nervous and lymphoid tissues. Recovered cats thus become latently infected carriers, and viral transmission (oronasal route) may then be associated with the reactivation of latency due to stress or other conditions. Infected cats show sneezing, excessive salivation, serous to mucopurulent ocular and nasal discharge, corneal ulcers, and severe conjunctivitis leading to chemosis [20,21,22,23,24].

Feline calicivirus (FCV) is a non-enveloped, single-stranded RNA virus of the family *Caliciviridae*, genus *Vesivirus*, characterized by significant genetic variability with many different strains varying slightly in antigenicity and pathogenicity. It is moderately resistant in the environment; although infected cats are the most important source of infection, environment persistence and carrier cats can also contribute to virus transmission (oronasal route). In addition to genetic variability, there is also a degree of biological variability responsible for various clinical signs ranging from respiratory symptoms, chronic stomatitis, and characteristic oral ulcers to a rare lethal presentation known as Virulent Systemic Disease (VSD) [21,23,25,26,27,28].

Core vaccines are designed for these three very dangerous, highly contagious, widespread, and often fatal diseases and are therefore strongly recommended for all cats regardless of their lifestyle or location. Each one of them should be vaccinated with core vaccines at least once in their life for a dual purpose: to prevent individual infections and/or ameliorate clinical disease and to try to guarantee herd immunity [6,29].

The ABCD has also proposed another category of vaccines, the so-called circumstantial ones [4]. This is the case for some non-core vaccines which, in certain situations and cases, can be reclassified as core vaccines and thus recommended for all cats, such as rabies and FeLV vaccines [4,29,30].

Vaccinations with core and non-core vaccines have traditionally been performed for decades on an annual basis. Current knowledge and newly available vaccines have led to the aforementioned international vaccination guidelines and experts suggesting changes by opting to vaccinate less frequently and by using modified live (attenuated) core vaccines (MLV) or killed core vaccines registered for triennial use [31]. However, while for dogs, the advice is to vaccinate adult animals no more frequently than every 3 years for all three core vaccines (parvovirus infection, distemper, and infectious canine hepatitis) [6,32,33,34], cats are not all the same, and neither are vaccines. In fact, FPV vaccines induce a very strong and long-lasting protective immune response, whereas FeHV-1 and FCV vaccines do not always provide the same protection degree, and their real duration of immunity (DOI) can be difficult to identify. Since protection seems to decrease with time, it is advisable to vaccinate cats with a tailored approach according to their lifestyle [2,4,5,6,24,26,29]. Cats can, in fact, be classified as low- or high-risk. Low-risk cats are generally solitary indoor cats that never visit a cat show, a groomer, or a boarding cattery, while high-risk cats live in an indoor/outdoor multicat household or regularly travel or visit feline exhibitions or boarding catteries. Consequently, high-risk cats should be vaccinated more frequently than low-risk cats, generally every 1–2 years rather than every 3 years, depending on the risk for each single cat [2,5,6,35].

In any case and for all vaccines, many factors interfere with the mounting of adequate immune protection; firstly, the interference of Maternally Derived Antibodies (MDAs) in kittens, as with puppies [2,6,29,36,37,38]. Therefore, knowing a kitten’s antibody protection could help to reduce both vaccination failures and unnecessary vaccinations. Moreover, antibody titration could also help identify the true condition of elderly cats and vaccinate those that are no longer protected, of course always considering possible different responses to FPV compared with FeHV-1 and FCV [39,40,41,42].

Consequently, knowing the true immune status of each cat with respect to core vaccines could help veterinarians choose the best vaccine protocol for each feline patient. Although the use of in-clinic tests for this purpose in dogs is gaining popularity in the pet veterinary world [6,34], their use in cats is still very limited, and there are only a few publications in the literature related to the evaluation of the specific immune response toward feline core vaccines.

The aim of this study was to determine the actual seroprotection against FPV, FeHV-1, and FCV in a large cohort of Italian cats by using an in-practice test kit.

## 2. Materials and Methods

### 2.1. Study Population and Study Protocol

The feline plasma and serum samples analyzed in this study were collected over 6 years (from June 2017 to June 2023) to both evaluate specific antibody titers and for other purposes (i.e., FIV/FeLV screening). According to the decision of the Ethical Committee of the University of Milan, residual aliquots of samples collected with the informed consent of owners can be used for research purposes without any additional formal request for authorization (EC decision, 29 October 2012; renewed with protocol n. 02-2016). For each cat, key information was always recorded: (1) the cat’s origin, owned or stray; (2) sex and reproductive status, intact or neutered female or male; (3) age according to the recent AAHA/AAFP guidelines on feline life stages [43], kittens and juveniles (simplified kittens) from 16 weeks of age (after the end of the eventual first vaccination series) to less than 1 year of age, young adults (from 1 to less than 7 years), mature adults (from 7 to less than 10 years), and seniors (10 years and older); (4) breed (common European or pure breed); (5) FIV/FeLV status; (6) health status, healthy or unhealthy considering only those clinical problems that could impact immune function; and (7) vaccination history, including the time elapsed since the last vaccination, ≤1 year, >1 year–≤3 years, or >3 years.

### 2.2. Detection of Specific Antibodies via VacciCheck

Each blood sample was assayed using the in-clinic test Feline VacciCheck (produced by Biogal, Kibbutz Galed, Israel, and supplied in Italy by Agrolabo, Scarmagno, Italy), following the manufacturer’s instructions. The kit is a rapid, semiquantitative dot-ELISA-based system licensed to determine antibody titers against FPV, FeHV-1, and FCV. The test was previously validated, showing good values of specificity (89%, 93%, and 90%) and sensitivity (98%, 96%, and 91%) for FPV, FeHV-1, and FCV, respectively [44]. This test can be applied in practice with limitations related to both respiratory viruses (FeHV-1 and FCV), as indicated in the WSAVA guidelines and many other studies [4,6,40,41,45,46].

With this test, antibody concentration is defined by the color intensity of the resulting spots compared with the “S” units on a scale from 1 to 6. An S value of 0 (S0) was standardized by the manufacturer as being equivalent to an antibody titer of <1:20 for FPV, <1:4 for FeHV-1, and <1:8 for FCV. An S value of 3 (S3) was standardized by the manufacturer to be equivalent to 1:80 for FPV, 1:16 for FeHV-1, and 1:32 for FCV (Table S1). Antibody titers equal to or higher than S3 values were considered indicative of a significant positive response, representing specific protection against these three viruses. Results were divided into four categories (unprotected, weakly positive, medium positive, and high positive) based on the threshold values of each pathogen (Table S2). Cats with antibody titers equal to the threshold value were considered medium positive. Medium-to-high positive results were expressed as Protective Antibody Titers (PATs).

### 2.3. Statistical Analysis

Statistical analyses were performed using GraphPad Prism 9 (La Jolla, CA, USA), considering statistically significant values at *p* < 0.05. A chi-square (χ^2^) analysis was used to determine significant differences between protected and unprotected cats. Antibody titer data were transformed using log_2_. The Shapiro–Wilk test was used to verify the normal distribution of data, and non-parametric Kruskal–Wallis and Mann–Whitney tests were used.

## 3. Results

### 3.1. Cat Population

A total of 740 feline serum/plasma samples were included in the study (Table 1). Of these, 567 were from owned cats (76.6%) and 173 were from stray (colony or shelter) cats (23.4%). Of the 740 cats analyzed, 352 (47.6%) were females (146 sexually intact and 206 neutered) and 388 (52.4%) were males (133 sexually intact and 255 neutered). Collectively, 279 cats (37.7%) were intact while 461 (62.3%) were neutered. The age ranged from 4 months to 25 years, with 109 kittens (14.7%), 352 young adults (47.6%), 112 mature adults (15.1%), and 167 seniors (22.6%). Considering breed, 643 cats were common European cats (86.9%), and only 97 were of pure breed (13.1%); the most representative breeds were Maine Coon (15, 2.0%), followed by Ragdoll (12, 1.6%), Siamese (12, 1.6%), British (11, 1.5%), Siberian (10, 1.4%), Chartreux (9, 1.2%) and Persian (9, 1.2%). Considering FIV/FeLV status, half of the cats (366, 49.5%) were tested for these retroviruses: 314 (85.8%) were negative, 17 (4.6%) were FIV-positive, 22 (6.0%) were FeLV-positive, and 13 (3.6%) were positive for both viruses. Furthermore, 551 cats were healthy (74.5%), while 189 (25.5%) had one or more clinical problems that could impact immune function (especially Chronic Kidney Disease (CKD), lymphadenopathy, hyperthyroidism, lymphoma, and other neoplasms). Finally, just over half of the cats (435, 58.8%) had been vaccinated almost once in their life (from 1 month to more than 18 years before sample collection), while 305 (41.2%) were unvaccinated. Among the 435 vaccinated cats, 170 (39.1%) were vaccinated ≤1 year before sampling, 150 (34.5%) received their last vaccination >1–≤3 years earlier, and 115 (26.4%) were vaccinated more than 3 years earlier. None of the shelter/colony cats were vaccinated.

### 3.2. Antibody Titers and Protection of the Entire Cohort

Specific antibody titers ranged from <1:20 to >1:640 for FPV, from <1:4 to >1:128 for FeHV-1, and from <1:8 to >1:256 for FCV. Specific PATs for FPV, FeHV-1, and FCV were observed in 504 (68.1%), 363 (49.1%), and 582 (78.6%) cats of the entire population of 740 cats, respectively. These percentages were even higher when considering only the 435 vaccinated cats (84.6%, 57.9%, and 82.3%, respectively). The distribution of PATs in the three feline populations considered (all 740 cats, 435 vaccinated cats, and 305 unvaccinated cats) divided into categories (origin, sex and reproductive status, age, breed, FIV/FeLV status, health status, and time elapsed since last vaccination, when applicable) is shown in Table 1 and in Figure S1.

**Figure 1:**
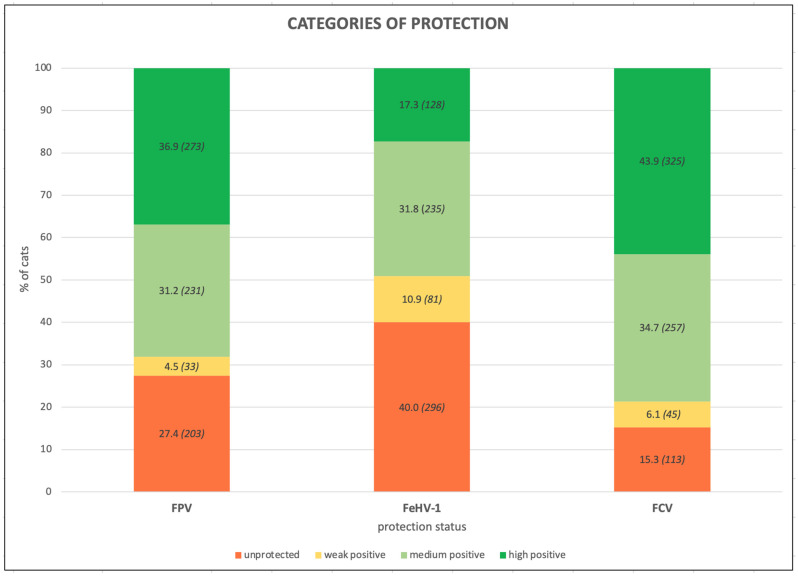
Percentages of cats (number of cats) belonging to the different categories of protection against Feline panleukopenia virus (FPV), Felid herpesvirus type 1 (FeHV-1), and Feline calicivirus (FCV) of the whole feline population (740 Italian cats).

The results were then divided into categories of protection (unprotected, weakly positive, medium positive, and high positive) based on the threshold values of each pathogen (Figure 1).

**Figure 2:**
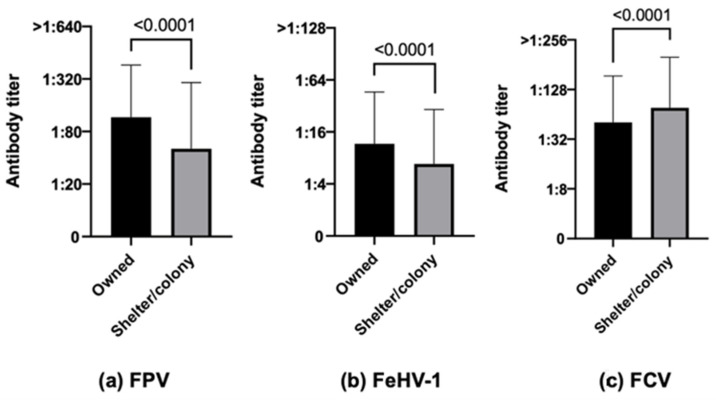
Antibody titers against Feline panleukopenia virus (FPV), Felid herpesvirus type 1 (FeHV-1), and Feline calicivirus (FCV), considering the variable origin, of the 740 Italian cats (Mann–Whitney test): 567 owned vs. 173 stray (colony/shelter) cats.

**Figure 3:**
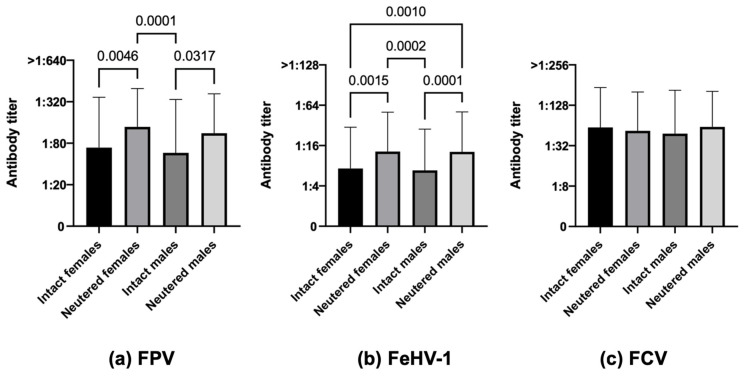
Antibody titers against Feline panleukopenia virus (FPV), Felid herpesvirus type 1 (FeHV-1), and Feline calicivirus (FCV), considering the variable sex and the reproductive status of the 740 Italian cats (Kruskal–Wallis test): 146 intact females vs. 206 neutered females vs. 133 intact males vs. 255 neutered males.

**Figure 4:**
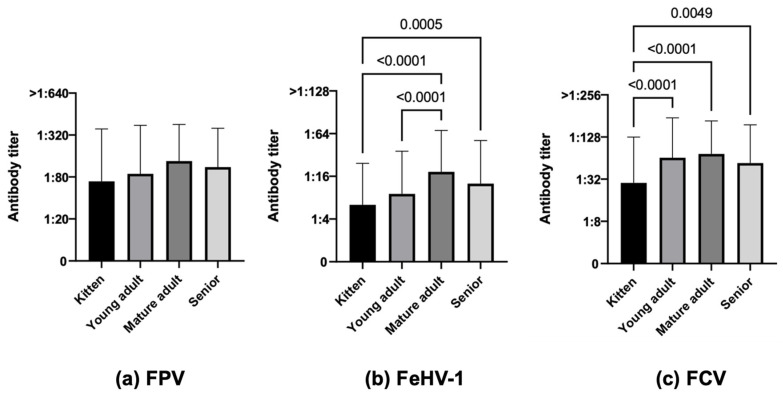
Antibody titers against Feline panleukopenia virus (FPV), Felid herpesvirus type 1 (FeHV-1), and Feline calicivirus (FCV), considering the variable age, of the 740 Italian cats (Kruskal–Wallis test): 109 kittens vs. 352 young adults vs. 112 mature adults vs. 167 seniors.

**Figure 5:**
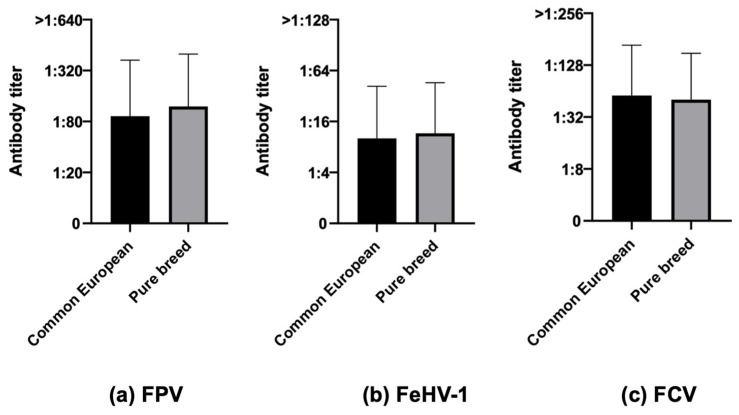
Antibody titers against Feline panleukopenia virus (FPV), Felid herpesvirus type 1 (FeHV-1), and Feline calicivirus (FCV), considering the variable breed, of the 740 Italian cats (Mann–Whitney test): 643 common European vs. 97 pure breed.

**Figure 6:**
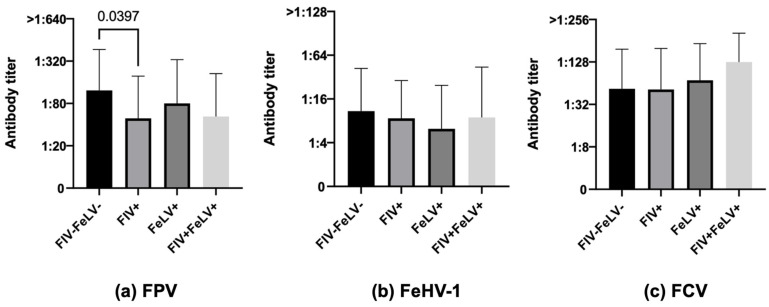
Antibody titers against Feline panleukopenia virus (FPV), Felid herpesvirus type 1 (FeHV-1), and Feline calicivirus (FCV), considering the variable FIV/FeLV status, of the 366 Italian cats tested for this condition (Kruskal–Wallis test): 314 double negatives vs. 17 FIV-positive vs. 22 FeLV-positive vs. 13 double-positive cats.

**Figure 7:**
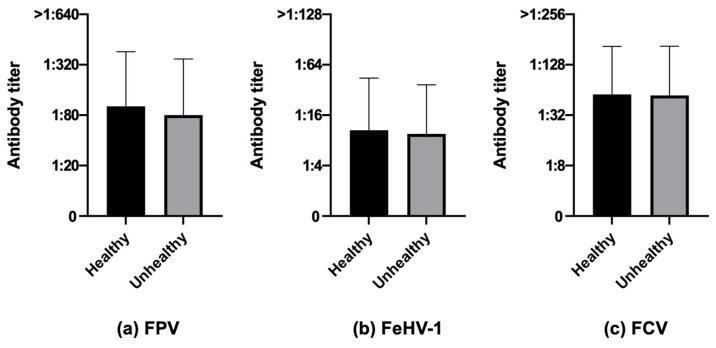
Antibody titers against Feline panleukopenia virus (FPV), Felid herpesvirus type 1 (FeHV-1), and Feline calicivirus (FCV), considering the variable health status, of the 740 Italian cats (Mann–Whitney test): 551 healthy cats vs. 189 unhealthy cats.

**Figure 8:**
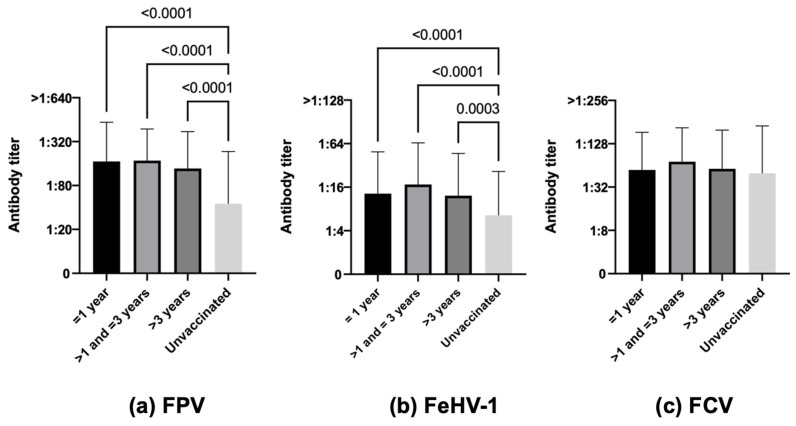
Antibody titers against Feline panleukopenia virus (FPV), Felid herpesvirus type 1 (FeHV-1), and Feline calicivirus (FCV), considering the variable vaccination, of the 740 Italian cats (Kruskal–Wallis test): 170 vaccinated ≤1 year before vs. 150 vaccinated >1–≤3 years before vs. 115 vaccinated >3 years before vs. 305 unvaccinated.

Table 2 refers to the results of the chi-square test applied to the 740 Italian cats, and Figure 2, Figure 3, Figure 4, Figure 5, Figure 6, Figure 7 and Figure 8 show the statistical results related to the whole feline population in this study (see below).

Table 3 reports the cats resulted seronegative (no antibodies) for one or more viruses.

Less than half of the entire cohort of 740 cats (36.4%, 269 cats) had good protection (PATs equal to or higher than the threshold values) against all three infectious agents simultaneously. This percentage increased if titers just below the threshold values (weak positive) were also considered (48.6%, 360 cats) and increased even more when considering only the 435 vaccinated cats (50.3%, 219 cats).

Only less than 20% of the 305 unvaccinated cats had PATs for all three diseases simultaneously (16.4%, 50 cats), but this percentage increased considering each disease (FPV, 44.6%; FeHV-1, 36.4%; and FCV, 73.4%) (data not present in the table).

The results of our study largely confirm the percentages of protection obtained in other studies worldwide (Table 4) which reported antibody titers measured in owned and/or stray cats using gold standard tests (Hemagglutination Inhibition (HI), Virus Neutralization (VN), and ELISA) or by in-clinic tests.

### 3.3. Results According to the Different Variables

#### 3.3.1. Origin

The results for FPV showed that owned cats were numerically more protected (75.0% owned cats vs. 45.7% stray cats, χ^2^ *p*-value < 0.0001) and had statistically higher antibody titers than stray (colony/shelter) cats (Figure 2a, *p*-value < 0.0001). Similar results characterized FeHV-1 (52.6% owned cats vs. 37.6% stray cats, χ^2^ *p*-value 0.0007, Figure 2b, *p*-value < 0.0001). Opposite results were, however, obtained regarding antibody titers for FCV, which were statistically higher in stray cats than in owned ones (Figure 2c, *p*-value < 0.0001).

#### 3.3.2. Sex and Reproductive Status

Considering FPV, neutered cats (both females and males) had a significatively greater number of protected individuals than intact ones (81.1% neutered females and 74.5% neutered males vs. 55.5% intact females and 49.6% intact males, χ^2^ *p*-value < 0.0001) and also had statistically higher antibody titers (Figure 3a, *p*-value 0.0046 intact females vs. neutered females, *p*-value 0.0001 neutered females vs. intact males, *p*-value 0.0317 intact males vs. neutered males). The same situation was observed for FeHV-1 (57.3% neutered females and 55.7% neutered males vs. 37.0% intact females and 36.8% intact males, χ^2^ *p*-value < 0.0001, Figure 3b, *p*-value 0.0015 intact females vs. neutered females, *p*-value 0.0002 neutered females vs. intact males, *p*-value 0.0001 intact males vs. neutered males, *p*-value 0.0010 intact females vs. neutered males). Regarding FCV, antibody differences were not statistically significant (Figure 3c).

#### 3.3.3. Age

When comparing age groups, kittens were the least protected age category, followed by young adults for all diseases in a statistically significant way both in terms of numbers and antibody titers for FeHV-1 (33.9% kittens vs. 44.0% young adults, 69.6% mature adults and 55.7% seniors, χ^2^ *p*-value < 0.0001, Figure 4b, *p*-value < 0.0001 kittens vs. mature adults, *p*-value 0.0005 kittens vs. seniors, *p*-value < 0.0001 young adults vs. mature adults) and for FCV, in this case with higher values (58.7% kittens vs. 81.0% young adults, 88.4% mature adults and 80.2% seniors, χ^2^ *p*-value < 0.0001, Figure 4c, *p*-value < 0.0001 kittens vs. young adults, *p*-value < 0.0001 kittens vs. mature adults, *p*-value 0.0049 kittens vs. seniors). For FPV, only the number of protected cats was significantly different between age categories due to a lower number of protected kittens (55.0% kittens vs. 64.8% young adults, 81.4% mature adults and 75.4% seniors, χ^2^ *p*-value < 0.0001) but not in terms of antibody titers (Figure 4a).

#### 3.3.4. Breed

Considering breed, no statistically significant differences were found between common European and pure breed cats for numbers nor antibody titers (Figure 5).

#### 3.3.5. FIV/FeLV Status

In the 366 cats tested for their FIV and FeLV status, cats negative for both retroviruses were more protected than FIV-positive ones in terms of both numbers and antibody titers but only for FPV (76.1% FIV–FeLV– vs. 47.1% FIV+, 68.2% FeLV+, 61.5% FIV+FeLV+, χ^2^ *p*-value 0.0364, Figure 6a, FIV–FeLV– vs. FIV+, *p*-value 0.0397). Similarly, statistically significant differences were observed for neither FeHV-1 nor for FCV for numbers of cats and antibody titers (Figure 6b,c).

#### 3.3.6. Health Status

When considering health status, no statistically significant differences could be noticed for neither the number of cats nor for antibody titers (Figure 7).

#### 3.3.7. Time Elapsed since Last Vaccination

Core protection was higher in vaccinated cats than in unvaccinated ones in terms of both the number of protected cats and antibody titers for FPV (83.5% vaccinated ≤1 year, 88.0% vaccinated >1–≤3 years, 81.7% vaccinated >3 years vs. 44.6% unvaccinated, χ^2^ *p*-value < 0.0001, Figure 8a, *p*-value < 0.001 vaccinated ≤1 year vs. unvaccinated, *p*-value < 0.001 vaccinated >1–≤3 years vs. unvaccinated, *p*-value < 0.001 vaccinated >3 years vs. unvaccinated) and for FeHV-1 (55.3% vaccinated ≤1 year, 62.6% vaccinated >1–≤3 years, 55.7% vaccinated >3 years vs. 36.4% unvaccinated, χ^2^ *p*-value < 0.0001, Figure 8b, *p*-value < 0.001 vaccinated ≤1 year vs. unvaccinated, *p*-value < 0.001 vaccinated >1–≤3 years vs. unvaccinated, *p*-value 0.003 vaccinated >3 years vs. unvaccinated). For FCV, the difference was statistically significant only for the number of protected cats (81.2% vaccinated ≤1 year, 84.7% vaccinated >1–≤3 years, 81.0% vaccinated >3 years vs. 73.4% unvaccinated, χ^2^ *p*-value < 0.0001) but not for antibody titers (Figure 8c). The antibody titers of 115 cats vaccinated at least more than 3 years before sampling are reported in Figure S2.

## 4. Discussion

According to the latest demographic analysis, the global cat pet population ranges from 300 to 600 million, and their number is increasing with time [66]. Owners’ attention to the care and well-being of their pets has also increased over time, and by now, many cat owners regularly visit a veterinarian to check their pets’ health status and to protect them against dangerous diseases through vaccination.

International vaccination guidelines all agree that vaccination is a vital practice for cats and that they should be regularly vaccinated against the major and most prevalent feline infectious diseases. For dogs, the three-year use basis for core vaccinations was defined because very effective long-lasting immunity is stimulated in most vaccinated dogs and consequently, it would be ethically and scientifically incorrect to opt for closer boosters [6,7,8,29,32,33,34,67,68,69]. For cats, however, the situation is slightly different.

In this study, cats tested positive primarily for FCV in terms of both number and antibody titers, and this was true for both owned and especially stray cats. This result agrees with the few available studies in the literature which report the highest positivity almost always involving FCV (see Table 4). Apart from being a core vaccine, FCV is ubiquitous in both urban and rural areas in Italy and abroad, and this is due to different causes. Its high resistance in the environment is probably the major contributor to its wide diffusion, along with its easy airborne and direct transmission via oronasal and conjunctival secretions and the carrier status of some infected cats [25,70]. All these features can be responsible for the strong circulation of the virus in the feline population, together with virus excretion by cats after infection and episodically after vaccination with MLV FCV vaccines, especially in colony cats which can be infected with distinct viruses evolving from distinct ancestors [29,70,71].

After FCV, the highest positivity is observed for FPV. Again, this is not unexpected since the pathogen is a parvovirus and thus, like its canine counterpart (CPV-2), it is highly immunogenic and highly resistant in the environment [13,16,17,18,19], thus contributing to both the maintenance and spread of the disease and the particularly high seroprotection rates. Indeed, in this study, 87.7% (442/504) of cats with PATs against FPV had antibody titers ≥1:160, and about half of them were categorized as high positive (titers ≥1:320). Furthermore, cats can also be infected with different variants of the canine parvovirus (CPV-2a, 2b, or 2c), causing panleukopenia only rarely (5% of cases) [72,73] but shedding in their feces [14,15,74,75,76], representing a potential source of environmental contamination for other cats. CPV-2-infected cats can produce antibodies indistinguishable from those against FPV. Although rare, this should be considered in countries in which the number of stray cats (colony or shelter ones) is quite high and where CPV-2 is quite common in the canine population, as is the case in Italy [64]. Conversely, FPV can also infect dogs, but they do not appear to shed the virus in the environment, thus posing no risk to cats [19].

Finally, the lowest PAT values for both animal numbers and antibody amounts were those for FeHV-1. Also, in this case, this should not be surprising, especially considering the characteristics of the virus. Differently from the other two viruses, the presence of an envelope makes this virus labile in the environment and easily eliminated by adverse climatic conditions and common disinfectants; consequently, transmission occurs primarily through close contact (fomites are important only in crowded environments) and by the recrudescence of latent FeHV-1 infection, with re-expressed viral proteins boosting immunity. In the field, natural boosters resulting from contact with infected cats might not occur frequently.

Although partial, protection against FeHV-1 can persist for several years after vaccination. However, the level of protection will decrease over time in cats that have not had a natural (i.e., exposure to field virus) or vaccine booster [13,19]. Apart from some exceptions, FeHV-1 represents the virus with the lowest number of seropositive cats in many studies (see Table 4).

Regarding FPV and FeHV-1, this study has shown that owned cats exhibit numerically and quantitatively higher protection compared to stray cats. Conversely, the opposite results were observed for FCV, for which PATs were statistically higher in stray cats than in owned ones. The higher protection observed for FPV in owned cats could be attributed, as previously mentioned, to the widespread prevalence of this resistant pathogen in the environment, coupled with the extensive FPV vaccination. Additionally, the severity of FPV infection in free-roaming cats lacking veterinary care might lead to fatal outcomes, thereby evading sampling and testing. The stray cats examined in our study were less protected from FeHV-1 compared to owned ones, likely due to the expected lower circulation of this pathogen in the considered areas according to the previously mentioned intrinsic characteristics. The higher FCV PATs in stray cats align with our previous findings [64]. Additionally, several other studies (see Table 4) consistently report this virus as widely circulating in the field, eliciting a strong immune response in cats.

Considering sex and reproductive status, in this study, neutered cats were more protected than intact ones for FPV and FeHV-1 but not for FCV. Of the very few papers considering this factor, the results of DiGangi et al. [59] showing neutered shelter cats more protected than intact ones for all three viruses agree with our findings. Other papers, however, have generally reported males as more protected than females [48,51,53] or no influence of the sex factor [49,61,77].

In this study, kittens and young adults were, in general, the two least protected categories both in terms of numbers and antibody titers for all three pathogens. Different age categories, namely young adults, adults, or seniors (but never kittens), have been reported as the most protected by different studies [53,55,57,59,63,64,65]. The apparent discrepancy concerning young adults (less protected according to our study, more protected according to others) could be attributed to the different criteria for defining feline age categories across the different studies. The “young adults” category in our work (cats from 1 to less than 7 years, following the indications of the recent AAHA/AAFP guidelines on feline life stages [43]), includes animals that are often classified as adults (>2 years old cats) in other studies. For further studies, the age factor unexpectedly did not influence protection [49,61]. The lower protection of kittens may be related to interference by MDA, which are considered the main cause of vaccine failure in young pets [29,36,37,63]. To bypass MDA interference, all international guidelines and experts agree to propose multiple vaccinations in kittens until at least 16 weeks of age or older [2,4,6,29]. This MDA interference can also explain why adult cats with an incomplete first vaccination protocol (i.e., only two vaccine administrations and/or vaccinations stopped at 12 weeks of age) sometimes fail to develop a correct humoral immune response.

Older cats can still be susceptible to pathogens since their immune system may struggle to mount a valid response to antigens they have never encountered before (primary immune response). However, they can continue to efficiently fight known antigens (secondary immune response), as is the case with vaccine boosters. It is therefore strongly recommended that vaccinations continue with an appropriate protocol throughout the cat’s life. This decision, however, will probably need to be explained to the owner, who will mistakenly believe that his/her cat is too old for vaccination [78].

When considering breed, the lack of statistically significant differences observed in this study aligns with the findings of Mende et al. [61], who examined common European and Maine Coon cats. It is noteworthy that breed has been identified as a significant factor for the response to vaccination in only one study and solely for FPV [62].

About half of the feline cohort in our study (366 cats) were tested for their FIV/FeLV status, demonstrating that negative cats were statistically more protected than FIV-positive ones in terms of both numbers and antibody titers, but only for FPV. Only a few other studies considered FIV/FeLV status as a factor affecting vaccine protection against other infectious diseases, demonstrating a significant association between them [49,79]. The efficacy of vaccines in immunocompromised cats seems to depend on the stage of FIV infection. Cats in an early stage of infection can mount good levels of protective antibodies after vaccination, while during the terminal phases, the immune response can be impaired. Probably, the FIV-infected cats of the two studies were in different phases of infection and thus able to mount different immune responses to vaccination.

When considering health status in this study, no statistically significant differences were observed, in accordance with previously published studies in shelter cats [59]. This is another factor which is unfortunately neglected in studies investigating cats’ protection against diseases which are preventable by core vaccines. Chronic Kidney Disease (CKD) has been associated with a lack of humoral immunity against FPV in cats [61]. An association between CKD and a reduced antibody response after vaccination has been documented in humans for hepatitis B [80,81]. Furthermore, malnutrition in patients with CKD has been shown to impair the immune response [82]. In our study, none of the 21 cats suffering from CKD had impaired immunity to any of the three considered pathogens, but this result may be due to the low number of cats with CKD analyzed. It will be interesting to investigate if frequent or annual vaccination may be a risk factor for the development of azotemic CKD in geriatric cats, as proposed by Finch et al. [83].

Neoplasms have also been associated with a lack of antibodies in cats [61], and the authors regretfully point out the absence in veterinary medicine of information on responses to vaccination in cancer patients. A meta-analysis in humans indicated lower rates of antibody development after vaccination in tumor patients [84]. In our study, more than 20 cats had lymphomas or other neoplasms, and none were receiving chemotherapy; of these, the only ones partially unprotected were three unvaccinated cats. Very recently, our research group published an interesting paper on the effect of chemotherapy on core vaccination response in canine oncologic patients [85], suggesting that contrary to expectations, chemotherapy does not have a marked immunosuppressive effect on the post-core vaccine antibody response in canine cancer patients, thus helping veterinarians better manage their patients and helping owners feel more confident about their pets’ life quality. It will be interesting to monitor whether such a positive result will also be maintained in canine and feline cancer patients not receiving chemotherapy (study in progress).

Finally, considering vaccination, vaccinated cats were numerically and quantitatively more protected than unvaccinated ones, and differences between vaccinated and unvaccinated cats were always statistically significant regardless of the time since the last vaccination for FPV and FeHV-1 but not for FCV. Considering FCV, antibody levels were quite similar irrespective of vaccination and were always protective. Most cats remained protected for up to 3 years after vaccination for FPV and FCV but less for FeHV-1, even if some studies have demonstrated that the FeHV-1 antibodies produced by memory cells generated during primary responses tend to slowly increase with time and are more common in older cats [65,77]. More specifically, as age advances, antibody titers for FPV remain at protective levels for up to 9 years after the last vaccination, after which they decrease to values below the threshold value (a statistically significant difference). For FCV, they decrease slowly over the years but always remain above the threshold value. In the case of FeHV-1, they exhibit a trend similar to FPV (a statistically significant difference) but with much lower values, approaching the threshold value as early as 3–5 years after the last vaccination (Figure S2). Our results further reinforce the persistence of long-lasting protective immunity, which can be measured many years after the last vaccination (in this study, up to more than 18 years later in the oldest cats). As already mentioned, other studies have also demonstrated that cats maintain sufficiently high PATs three or more years after their last vaccination, above all for FPV [32,68,69,86]. Nevertheless, since not all cats are protected after so many years and for all pathogens, checking protection using antibody titration could be the best choice to prevent immunity breakdowns.

### Reliability and Usefulness of Assessment of Antibody Titration for Cat Core Vaccines in Practice

The use of antibody titration to estimate protection before or after a core vaccination should be the first choice of every veterinary practitioner in daily practice to properly vaccinate their patients. This has always been possible through the use of gold standard tests (HI and VN), while only in recent years have practical in-clinic tests become commercially available such as the VacciCheck, which was used in this study and in similar studies of ours when core vaccine protection in dogs was the focus [67,85,87,88,89,90]. Antibody titer tests may be very useful for monitoring immunity specific to core vaccines through a careful interpretation of antibody titration results [67].

The use of antibody titration to determine whether or not a cat is protected against FPV is considered useful for personalized medicine and tailored vaccination. Titrating instead of merely vaccinating a potentially protected young, adult, or elderly cat may in fact prevent overvaccination. Conversely, if titration fails to yield the expected result, it allows for a timely intervention to vaccinate a cat that was believed to be protected. Having confirmed the usefulness of antibody titration for FPV, a universally agreed-upon protective titer for adult cats remains undetermined, ranging from 1:80 (as used in this study) to lower antibody titers such as 1:20 as even this low titer indicates an immune response to an antigen [6,35,52,60,91,92,93]. When an antigen is highly immunogenic, as is the case with a parvovirus (FPV), it is reasonable to expect high antibody titers in protected animals. Conversely, titers below the conventional 1:80 threshold raise concerns among veterinarians as they may indicate unsuccessful stimulation. The same WSAVA guidelines and many published studies reported that antibody titers perfectly correlate with protection [6,33,41,94].

The scientific community universally accepts the reliability of in-clinic tests for canine core vaccines and feline panleukopenia vaccines, making their use recommended. However, there is still considerable debate about their accuracy in evaluating protection against feline upper respiratory diseases caused by FeHV-1 and FCV. Indeed, many authors underline that in this case, the effectiveness of antibody testing in predicting protection and serving as a pre-vaccination index is limited. It is crucial to note that antibody titration can never replace routine vaccination against FeHV-1 and FCV. For FCV, one supported reason is that the antibodies detected may not provide protection against the specific FCV strains to which the cat will be exposed in the field [25,26,63]. In this scenario, it would be better to perform a Virus Neutralization (VN) test which is able to detect neutralizing antibodies that are powerful and thus useful in preventing infection. However, a VN may be less sensitive than an ELISA since it is strongly influenced by the antigenic relationship between the isolate used in the test and that of the vaccinated or infected cat [63]. For this reason, some researchers opt to use ELISA assays instead of (or in addition to) VN as they are not impacted by this potential issue [52,63]. VacciCheck is an in-clinic test based on a dot-ELISA test; thus, it should not have this problem. Consequently, it might be suitable for this purpose as well.

Conversely, a lack of serum antibodies in vaccinated cats does not necessarily indicate their susceptibility to a disease. A rapid immune response, associated with the reactivation of the memory cells undoubtedly present in a previously vaccinated animal, contributes to protection even if serum antibodies have diminished to low levels and become undetectable [6,35,95]. Furthermore, in the case of FCV infection, protection can be associated with robust cell-mediated and mucosal immunity (mucosal IgAs seem to be more strongly linked to protection than serum antibodies), but measuring either of them is neither easy nor routine [6,25,35,64,96,97].

The same problem may also characterize FeHV-1, for which failure to detect specific antibodies is reported in vaccinated cats. In a study by Lappin et al. [52], however, seronegative cats resisted a subsequent FeHV-1 challenge, demonstrating once again that other types of immunity may play an important role. Also, for this virus, robust cell-mediated immunity is even more important than humoral immunity, especially with MLV vaccines. Although some killed vaccines can still be effective and protective, primarily by stimulating the production of neutralizing antibodies, leading to a reduction in the duration of clinical signs and viral shedding [31]. However, measuring cell-mediated immunity requires sophisticated laboratory instruments [41,65,98].

The evidence suggests that protection is possible even when antibodies are not detectable, thus rendering antibody absence non-predictive of disease susceptibility. Therefore, some cats may be vaccinated even if already protected, but the incidence of this wrong choice would still be lower than the unnecessary vaccination of cats with an arbitrary booster [52]. This scenario can occur when a veterinarian opts to vaccinate blindly without prior antibody titration.

Lappin et al. [52] demonstrated that a virus-specific antibody titer is correlated with protection from challenge, suggesting that individual antibody titration could serve as an alternative to the conventional blind vaccination approach. Using an ELISA to detect specific antibodies in vaccinated cats can help tailor the best vaccine approach for each individual. The VacciCheck used in this study is a rapid test based on a dot-ELISA method. Detecting specific antibodies for FeHV-1 and FCV, as in the case of FPV, might indeed indicate whether cats are susceptible to the disease. Most vaccinated cats had detectable antibodies, and this suggests resistance to infection. In these cases, a booster vaccination would not be necessary. Some years ago, Scott and Geissinger [86], in a study on core-vaccinated and then challenged cats, were able to demonstrate that antibodies specific to all three viruses persisted at protective levels for more than 3 years and that protection against FPV was longer (7.5 years) than against FHV and FCV (3–4 years), thus suggesting abandoning the common practice of revaccinating all cats on an annual basis. A few years later, even Mouzin et al. [35,99] reiterated the well-known concept, introduced some years before by Tizard [95]. Although serum antibodies cannot neutralize viruses once they are inside cells, they still contribute to defenses and can be considered a valid index of cat immunity. This perspective supports the WSAVA’s recommendation of vaccinations on a three-year basis after the first annual booster. This suggestion was echoed by DiGangi in 2011 [58], arguing that the VacciCheck, with its high diagnostic accuracy, can be a practical tool for assessing PATs against FeHV-1 and FCV. However, one year later, the same author stated that because FCV and FeHV-1 infections often lead to chronic carriers and only provide partial immunity, interpreting antibody titers becomes less predictive of protection from clinical disease [37]. These concerns have prompted an in-depth debate over what are the best vaccination intervals for these respiratory viruses, whether one year or more. Many studies support a duration of protection of minimum 3 years after the end of the first vaccine series (including the booster one year later) in kittens; in fact, canine and feline core MLV vaccines (or killed vaccines registered for a triennial use [31]), when properly administered, provide years of protection following effective vaccination, obviously in the absence of maternal antibody interference [35,60,61,68,77,86,99,100,101].

In the personal experience of the first author of this study, based on several hundred samples of dogs and cats tested with VacciCheck over many years, it can be asserted that in animals with PATs for a core vaccine, vaccine failures have never been reported. Moreover, no particular difference in efficacy has ever been noted after the use of feline versus canine core vaccines.

It is really important to vaccinate kittens with repeated administrations of core vaccines until they reach 16 weeks of age or older, schedule a booster one year later (the closure of the first vaccine series), and then continue with vaccinations on a three-year basis unless epidemiological conditions suggest closer boosters (e.g., high-risk cats) and, when possible, control protection using in-clinic tests before vaccination. This practice avoids unnecessary vaccination, in this way limiting the risk of developing the dreaded feline injection site sarcoma (FISS) in cats [2,6,101,102]. All inflammatory reactions can lead to the development of FISS by triggering uncontrolled proliferation, especially of fibroblasts, and in some cases, this results in malignant transformation. The risk seems unusually high for vaccines compared with other injections. Among vaccines, it seems higher for adjuvanted ones and lower for MLV and recombinant ones, but none are risk-free [103,104]. Some help in limiting the problem may come from the recent reduced-volume feline core vaccines (which nevertheless maintain an identical vaccine dose). By limiting inflammation at the injection site, they also appear to reduce the risk of FISS [105].

It is always advisable to remember that vaccination is not synonymous with protection, and vaccines almost never protect 100% of the vaccinated population (neither in veterinary nor in human medicine). Our results showed a high percentage (43.9%) of cats testing negative for one or more antigens; among them, 50 cats (6.8%) were negative for all the three core vaccines (but only 12 of them were vaccinated, see Table 3), confirming the possible lack of protection in vaccinated cats (with the last vaccination administered within a period ranging from a few months to 18 years). This lack of protection in vaccinated cats has also been reported in other studies, and these animals were considered non-responders [62,63,65]. Our study shows the highest prevalence of apparently non-responder cats concerning FeHV-1 (at least in terms of humoral immunity, given the absence of specific antibodies), with 243 out of 740 cats (32.8%) negative for FeHV-1 alone or also to one or both other viruses, in agreement with previous findings [65]. The problem of non-responders is well known both in human and veterinary medicine, and in the latter, especially in particular breeds of dogs such as Rottweiler and Doberman Pinscher, is due to genetic features [6,29,34,67]. Apart from genetics, it is also possible in feline medicine, and reasons explaining these seronegative findings are different, such as chronic diseases, diabetes, obesity or, conversely, malnutrition and FIV or FeLV positivity. The number of cats in studies (ours and others in the literature) experiencing such problems, however, was so small that it could not justify such a result. Rather, incorrect vaccine storage, leading to the inactivation of MLV vaccines, could have also played a role [62,106]. This could be the case for the vaccine for FeHV-1, which is the only of the three core viruses to have an envelope and is thus more easily inactivated and no longer able to properly stimulate the immune system. It is also possible that the vaccines were neutralized by antibodies already present in the vaccinated cats at the time of the vaccine booster before they were able to stimulate the memory cells. This is a very useful reminder that a vaccine is unlikely to induce a booster effect when a cat with high specific immunity is revaccinated; as reported in the WSAVA guidelines, administering more frequent vaccines to animals in an attempt to increase antibody titers is a futile exercise [6]. Moreover, since VacciCheck has high but not 100% sensitivity for any of the three diseases (FPV, 98%; FeHV-1, 96%; FCV, 91%, see Table S1), some negative cats could actually represent false negatives. So, following the pet vaccination recommendations, a cat that is seronegative for one of the three core vaccines (true or false negative) should be revaccinated and then retested to check whether seroconversion has occurred or not. The problem could be the availability of monovalent or bivalent vaccines to boost only the valence that fails to immunize the cat. In Italy, as in many other countries, there are no monovalent feline core vaccines, while a single bivalent vaccine is available for the respiratory forms caused by FeHV-1 and FCV to be used when there is no need to boost panleukopenia as well [31].

Finally, almost half of all the cats in our study (305 out of 740, 41.2%) had never been vaccinated. Apart from the 173 stray (colony/shelter) cats, of which we supposed none were vaccinated, one-quarter of the owned cats (132 out of 567, 23.3%) had never received a vaccination in their life. Of these, 85 (64.3%) were kittens but old enough (at least 4 months) to have been allowed to complete the first vaccination series. This low propensity for cat vaccination probably reflects the thinking of some owners that cats represent a world apart and can live without special affection, attention, care, or vaccination. Nothing could be more wrong. Cats deserve the same treatment as dogs, and there is no reason to leave them to their fate. Luckily, many of the unvaccinated cats in our study were protected from all three diseases. The same protection has been reported also in other studies both in owned and non-owned cats (see Table 4).

This study, nevertheless, has some limitations. First of all, only half of the cat population was tested for FIV and FeLV. The identification of retrovirus-infected cats remains an important factor for preventing new infections, and the FIV/FeLV status of each cat should be known. Unfortunately, too many veterinarians do not offer this screening to their clients for all cats, and therefore, for all cats, the real retrovirus status and the possible impact of these infections on their immune systems and vaccinations are not known. Secondly, although the sample size of this study was quite large, it does not necessarily mean that the analyzed cats are representative of the entire feline population in Italy. For this reason, it would be very useful to continue this type of analysis on a larger number of subjects, thus also increasing the numerosity of the relative subgroups (i.e., origin, sex, age, breed, the aforementioned FIV/FeLV status, health status, and vaccination history). Thirdly, it has been repeatedly mentioned that the URTD viruses (field and vaccine ones), apart from humoral immunity, stimulate cell-mediated and mucosal immunity, and these latter types of immunity are most closely related to protection. Finally, feline VacciCheck has good but non-optimal sensitivity and specificity for any of the three viruses (see Table S1), with the possibility, though rare, of false-positive results (for non-optimal specificity), especially for FPV (89%), or false-negative results (for non-optimal sensitivity), especially for FCV. The gold standard tests (HI and VN) would then be preferable to the rapid tests, but they have many disadvantages, especially the difficult and time-consuming nature of assaying, low practicality, and the need for a specialized laboratory.

## 5. Conclusions

This study is the first to examine a large cohort of Italian cats, both owned and stray ones, revealing the presence in many of them of good specific immunity to the major and most important feline infectious diseases as a result of both core vaccination and natural infection. Given the characteristics of the viruses, as might be expected, the highest protection was found for FCV, and the lowest protection was found for FeHV-1. Since veterinarians today can check the actual immune status of each cat, it becomes more difficult for a vaccinated cat to become sick with one of these infectious agents from a breakdown in immunity. Many feline core vaccines are registered for all threes viruses with a 3-year duration of immunity derived from post-challenge protection studies, but it is still always worthwhile to consider possible closer vaccinations (every 1 to 2 years) in cats at a high risk due to their lifestyle, preceded, when possible, by an assessment of specific antibody titers, even if for FeHV-1 and FCV, cell-mediated immunity and mucosal IgAs, respectively, are more strongly correlated with protection. The decision to vaccinate, even with core vaccines, should be based on a careful assessment of the likelihood of exposure and the severity of disease, as well as the risk/benefit ratio for each disease and for each cat, for which the benefits of vaccination should always outweigh the risk of adverse reactions (especially FISS).

The feline in-clinic test used in this study, the only one present on the Italian market, seems to provide reliable and trustworthy results and confirms itself as a valid tool in everyday veterinary practice when deciding if core vaccine boosters are needed for a cat or whether they can be postponed to the following year or even later.

## Figures and Tables

**Table 1:** Percentages and numbers (in italics in brackets) of cats with Protective Antibody Titers (PATs) for Feline panleukopenia virus (FPV), Felid herpesvirus type 1 (FeHV-1), and Feline calicivirus (FCV) according to origin, sex and reproductive status, age, breed, FIV/FeLV status (when tested), and health status of the whole feline population (740 cats), vaccinated cats (435 cats, for which the time elapsed since the last vaccination is also reported), and unvaccinated cats (305 cats).

	Protective Antibody Titers (PATs), % (*n. of Cats*)								
	Whole Population (740 Cats)			Vaccinated Cats (435 Cats)			Unvaccinated Cats (305 Cats)		
	FPV	FeHV-1	FCV	FPV	FeHV-1	FCV	FPV	FeHV-1	FCV
Total Positive	**68.1** (*504/740*)	**49.1** (*363/740*)	**78.6** (*582/740*)	**84.6** (*368/435*)	**57.9** (*252/435*)	**82.3** (*358/435*)	**44.6** (*136/305*)	**36.4** (*111/305*)	**73.4** (*224/305*)
Origin									
Owned cats	**75.0** (*425/567*)	**52.6** (*297/567*)	**77.4** (*438/567*)	**84.6** (*368/435*)	**57.9** (*252/435*)	**82.3** (*358/435*)	**43.2** (*57/132*)	**34.8** (*46/132*)	**61.4** (*81/132*)
Shelter or colony cats	**45.7** (*79/173*)	**37.6** (*66/173*)	**82.7** (*144/173*)	**0** (*0/0*)	**0** (*0/0*)	**0** (*0/0*)	**45.7** (*79/173*)	**37.6** (*65/173*)	**82.7** (*143/173*)
Sex and reproductive status									
Intact females	**55.5** (*81/146*)	**37.0** (*54/146*)	**78.8** (*115/146*)	**84.8** (*39/46*)	**47.8** (*22/46*)	**73.9** (*34/46*)	**42.0** (*42/100*)	**32.0** (*32/100*)	**81.0** (*81/100*)
Neutered females	**81.1** (*167/206*)	**57.3** (*118/206*)	**80.1** (*165/206*)	**89.4** (*135/151*)	**60.9** (*92/151*)	**84.1** (*127/151*)	**58.2** (*32/55*	**47.3** (*26/55*)	**69.1** (*38/55*)
Intact males	**49.6** (*66/133*)	**36.8** (*49/133*)	**72.2** (*96/133*)	**80.4** (*37/46*)	**60.9** (*28/46*)	**82.6** (*38/46*)	**33.3** (*29/87*)	**24.1** (*21/87*)	**62.1** (*54/87*)
Neutered males	**74.5** (*190/255*)	**55.7** (*142/255*)	**80.8** (*206/255*)	**81.8** (*35/192*)	**57.3** (*110/192*)	**82.8** (*159/192*)	**52.4** (*33/63*)	**50.8** (*32/63*)	**74.6** (*47/63*)
Age									
Kittens (4 months–<1 year)	**55.0** (*60/109*)	**33.9** (*37/109*)	**58.7** (*64/109*)	**78.6** (*44/56*)	**44.6** (*25/56*)	**73.2** (*41/56*)	**30.2** (*16/53*)	**22.6** (*12/53*)	**43.4** (*23/53*)
Young adults (≥1–<7 years)	**64.8** (*228/352*)	**44.0** (*155/352*)	**81.0** (*285/352*)	**88.4** (*152/172*)	**54.7** (*94/172*)	**82.6** (*142/172*)	**42.2** (*76/180*)	**33.9** (*61/180*)	**79.4** (*143/180*)
Mature adults (≥7–<10 years)	**80.4** (*90/112*)	**69.6** (*78/112*)	**88.4** (*99/112*)	**87.0** (*67/77*)	**74.0** (*57/77*)	**90.9** (*70/77*)	**65.7** (*23/35*)	**60.0** (*21/35*)	**82.9** (*29/35*)
Seniors (≥10 years)	**75.4** (*126/167*)	**55.7** (*93/167*)	**80.2** (*134/167*)	**80.8** (*105/130*)	**58.5** (*76/130*)	**80.8** (*105/130*)	**56.8** (*21/37*)	**45.9** (*17/37*)	**78.4** (*29/37*)
Breed									
Common European	**67.5** (*434/643*)	**48.8** (*314/643*)	**78.7** (*506/643*)	**85.5** (*301/352*)	**58.2** (*205/352*)	**82.7** (*291/352*)	**45.7** (*133/291*)	**37.5** (*109/291*)	**73.9** (*215/291*)
Pure breed	**72.2** (*70/97*)	**50.5** (*49/97*)	**78.4** (*76/97*)	**80.7** (*67/83*)	**56.6** (*47/83*)	**80.7** (*67/83*)	**21.4** (*3/14*)	**14.3** (*2/14*)	**64.3** (*9/14*)
FIV/FeLV status *									
FIV–FeLV–	**76.1** (*239/314*)	**49.7** (*156/314*)	**77.7** (*244/314*)	**83.2** (*198/238*)	**53.8** (*128/238*)	**79.4** (*189/238*)	**53.9** (*41/76*)	**36.8** (*28/76*)	**72.4** (*55/76*)
FIV-positive	**47.1** (*8/17*)	**47.1** (*8/17*)	**82.4** (*14/17*)	**71.4** (*5/7*)	**42.9** (*3/7*)	**85.7** (*6/7*)	**30.0** (*3/10*)	**50.0** (*5/10*)	**80.0** (*8/10*)
FeLV-positive	**68.2** (*15/22*)	**31.8** (*7/22*)	**86.4** (*19/22*)	**70.6** (*12/17*)	**29.4** (*5/17*)	**88.2** (*15/17*)	**60.0** (*3/5*)	**40.0** (2*/5*)	**80.0** (*4/5*)
FIV- and FeLV-positive	**61.5** (*8/13*)	**38.5** (*5/13*)	**92.3** (*12/13*)	**83.3** (*5/6*)	**33.3** (*2/6*)	**100.0** (*6/6*)	**42.9** (3*/7*)	**42.9** (*3/7*)	**85.7** (*6/7*)
Health status									
Healthy	**70.1** (*386/551*)	**49.9** (*275/551*)	**78.9** (*435/551*)	**87.5** (*300/343*)	**62.1** (*213/343*)	**83.1** (*285/343*)	**41.3** (*86/208*)	**29.8** (*62/208*)	**72.1** (*150/208*)
Unhealthy	**62.4** (*118/189*)	**46.6** (*88/189*)	**77.8** (*147/189*)	**73.9** (*68/92*)	**42.4** (*39/92*)	**79.3** (*73/92*)	**51.5** (*50/97*)	**50.5** (*49/97*)	**76.3** (*74/97*)
Time after vaccination **									
≤1 year	//	//	//	**83.5** (*142/170*)	**55.3** (*94/170*)	**81.2** (*138/170*)	//	//	//
>1–≤3 years	//	//	//	**88.0** (*132/150*)	**62.6** (*94/150*)	**84.7** (*127/150*)	//	//	//
>3 years	//	//	//	**81.7** (*94/115*)	**55.7** (*64/115*)	**81.0** (*93/115*)	//	//	//

* This variable was calculated for the 366 tested cats; ** this variable was calculated for the 435 vaccinated cats.

**Table 2:** Percentages and numbers (in italics in brackets) of the chi-square test for Feline panleukopenia virus (FPV), Felid herpesvirus type 1 (FeHV-1), and Feline calicivirus (FCV) antibody protection according to origin, sex and reproductive status, age, breed, FIV/FeLV status (when tested), and the health status of the whole feline population (740 cats), the vaccinated cats (435 cats, for which the time elapsed since the last vaccination is also reported), and the unvaccinated cats (305 cats).

		FPV			FeHV-1			FCV		
		Protected % (*Number*)			Protected % (*Number*)			Protected % (*Number*)		
Statistical Variable (*Number*)		YES	NO	*p*-Value	YES	NO	*p*-Value	YES	NO	*p*-Value
Origin	Owned (*567*)	75.0 (*425*)	25.0 (*142*)	**<0.0001**	52.6 (*297*)	47.4 (*270*)	**0.0007**	77.4 (*438*)	22.6 (*129*)	0.1681
	Shelter/colony (*173*)	45.7 (*79*)	54.3 (*94*)		37.6 (*66*)	62.4 (*107*)		82.7 (*144*)	17.3 (*29*)	
Sex	Intact females (*146*)	55.5 (81)	44.5 (65)	**<0.0001**	37.0 (54)	63.0 (92)	**<0.0001**	78.8 (115)	21.2 (31)	0.2397
	Neutered females (*206*)	81.1 (167)	18.9 (39)		57.3 (118)	42.7 (88)		80.1 (165)	19.9 (41)	
	Intact males (*133*)	49.6 (66)	50.4 (67)		36.8 (49)	63.2 (84)		72.2 (96)	12.8 (37)	
	Neutered males (*255*)	74.5 (190)	25.5 (65)		55.7 (142)	44.3 (113)		80.8 (206)	19.3 (49)	
Age	Kittens (*109*)	55.0 (60)	45.0 (49)	**<0.0001**	33.9 (37)	66.1 (72)	**<0.0001**	58.7 (64)	41.3 (45)	**<0.0001**
	Young adults (*352*)	64.8 (228)	35.2 (124)		44.0 (155)	56.0 (197)		81.0 (285)	19.0 (67)	
	Mature adults (*112*)	81.4 (91)	18.9 (21)		69.6 (78)	30.6 (34)		88.4 (99)	11.7 (13)	
	Seniors (*167*)	75.4 (126)	24.6 (41)		55.7 (93)	44.3 (74)		80.2 (134)	19.8 (33)	
Breed	Common European (*643*)	67.5 (*434*)	32.5 (*209*)	0.4138	48.8 (*314*)	51.2 (*329*)	0.8276	78.7 (*506*)	21.3 (*137*)	0.9999
	Pure breed (*97*)	72.2 (*70*)	27.8 (*27*)		50.5 (*49*)	49.5 (*48*)		78.4 (*76*)	21.6 (*21*)	
FIV/FeLV status	FIV–FeLV– (*314*)	76.1 (*239*)	23.9 (*75*)	**0.0364**	49.7 (*156*)	50.3 (*158*)	0.2602	77.7 (*244*)	22.3 (*70*)	0.4691
	FIV+ (*17*)	47.1 (*8*)	52.9 (*9*)		47.1 (*8*)	52.9 (*9*)		82.4 (*14*)	17.6 (*3*)	
	FeLV+ (*22*)	68.2 (*15*)	31.8 (*7*)		31.8 (*7*)	68.2 (*15*)		86.4 (*19*)	13.6 (*3*)	
	FIV+FeLV+ (*13*)	61.5 (*8*)	38.5 (*5*)		38.5 (*5*)	61.5 (*8*)		92.3 (*12*)	7.7 (*1*)	
Health status	Healthy (*551*)	70.1 (*386*)	29.9 (*165*)	0.0712	49.9 (*275*)	50.1 (*276*)	0.4507	78.9 (*435*)	21.1 (*116*)	0.6822
	Unhealthy (*189*)	62.4 (*118*)	37.6 (*71*)		46.6 (*88*)	53.4 (*101*)		77.8 (*147*)	22.2 (*42*)	
Time after vaccination	≤1 year (*170*)	83.5 (*142*)	16.5 (*28*)	**<0.0001**	55.3 (*94*)	44.7 (*76*)	**<0.0001**	81.2 (*138*)	18.8 (*32*)	**0.0258**
	>1–≤3 years (*150*)	88.0 (*132*)	12.0 (*18*)		62.6 (*94*)	37.4 (*56*)		84.7 (*127*)	15.3 (*23*)	
	>3 years (*115*)	81.7 (*94*)	18.3 (*21*)		55.7 (*64*)	44.3 (*51*)		81.0 (*93*)	19.1 (*22*)	
	Unvaccinated (305)	44.6 (*136*)	55.4 (*169*)		36.4 (*111*)	63.6 (*194*)		73.4 (*224*)	26.6 (*81*)	

In bold, statistically significant *p*-values.

**Table 3:** Percentages (numbers) of cats seronegative for at least one among Feline Panleukopenia Virus (FPV), Feline Herpesvirus type 1 (FeHV-1), and Feline Calicivirus (FCV).

Seronegative Cats	Whole Population (740 Cats) % (*n*. of Cats)	Vaccinated Cats (435 Cats) % (*n*. of Cats)	Unvaccinated Cats (305 Cats) % (*n*. of Cats)
Only for FPV	8.4 (*62*)	3.2 (*14*)	15.7 (*48*)
Only for FeHV-1	13.2 (*98*)	12.2 (*53*)	14.8 (*45*)
Only for FCV	1.5 (*12*)	1.6 (*7*)	1.6 (*5*)
For FPV and FeHV-1	8.8 (*65*)	1.8 (*8*)	18.7 (*57*)
For FPV and FCV	1.1 (*8*)	0.2 (*1*)	2.3 (*7*)
For FeHV-1 and FCV	4.1 (*30*)	3.9 (*17*)	4.3 (*13*)
For FPV, FeHV-1 and FCV	6.8 (*50*)	2.8 (*12*)	12.5 *(38)*
**Total**	**43.9 (*325*)**	**25.7 (*112*)**	**69.8 (*213*)**
For FPV (alone or with one or both other viruses)	25.0 (*185*)	8.0 (*35*)	49.2 (*150*)
For FeHV-1 (alone or with one or both other viruses)	32.8 (*243*)	20.7 (*90*)	50.2 (*153*)
For FCV (alone or with one or both other viruses)	13.5 (*100*)	8.5 (*37*)	20.7 (*63*)

**Table 4:** Percentages of protection against Feline panleukopenia virus (FPV), Felid herpesvirus type 1 (FeHV-1), and Feline calicivirus (FCV) detected in different studies since the 1980s in the specific antibody titration of cat samples worldwide by means of gold standard tests or in-clinic test kits performed on owned or stray cats.

				% of Protection		
Authors (year)	Reference	Country	No. of Cats	FPV	FeHV-1	FCV
Coman et al. (1981)	[47]	Australia	* 300	79.0	11.0	77.0
Yagami et al. (1985)	[48]	Japan	** 507	//	20.1	81.3
Yamaguchi et al. (1996)	[49]	UK	* 45	96.0	100.0	100.0
Miyazawa et al. (1999)	[50]	Vietnam	* 69	53.6	1.4	39.1
Nakamura et al. (1999)	[51]	Vietnam	* 50	44.0	44.0	74.0
Lappin et al. (2002)	[52]	USA	276	68.5	70.7	92.4
Ostrowski et al. (2003)	[53]	Saudi Arabia	* 13	8.0	15.0	39.0
Mouzin et al. (2004)	[35]	USA	272	96.7	88.2	97.8
Fischer et al. (2007)	[54]	Florida (USA)	* 61	33.0	21.0	64.0
Levy et al. (2008)	[55]	Galapagos	52	67.0	10.0	44.0
Blanco et al. (2009)	[56]	Costa Rica	96	92.8	71.9	//
Hellard et al. (2011)	[57]	France	273	36.6	54.2	77.6
			* 219	15.9	67.8	86.6
DiGangi et al. (2011)	[58]	Florida (USA)	* 356	41.0	10.0	36.0
DiGangi et al. (2012)	[59]	Florida (USA)	* 347	39.8	11.0	36.6
Mende et al. (2014)	[60]	Germany	347	63.0	//	//
Mende et al. (2014)	[61]	Germany	350	70.6	//	//
Bergmann et al. (2018)	[62]	Germany	112	64.3	//	//
Bergmann et al. (2019)	[63]	Germany	111	//	//	° 62.2
						°° 77.2
Dall’Ara et al. (2019)	[64]	Italy	* 151	45.6	37.0	85.4
Bergmann et al. (2020)	[65]	Germany	110	//	40.9	//
Our study (2023)	//	Italy	567	75.0	52.6	77.4
			* 173	45.7	37.6	82.7

* Unvaccinated shelter, colony, or free-ranging/feral cats; ** experimental cats of various origins; ° results obtained via a virus-neutralization test (VN); °° results obtained via an ELISA.

## Data Availability

The authors confirm that the datasets analyzed during the study are available from the first author/corresponding author upon reasonable request.

## Supplementary Materials

The following supporting information can be downloaded at https://www.mdpi.com/article/10.3390/life13122249/s1, Table S1: VacciCheck: correspondence between S scale units and antibody titers, sensitivity, and specificity for Feline Panleukopenia Virus (FPV), Feline Herpesvirus type 1 (FHV-1), and Feline Calicivirus (FCV); Table S2: Classification of protection categories for Panleukopenia Virus (FPV), Feline Herpesvirus type 1 (FHV-1), and Feline Calicivirus (FCV) in 740 Italian cats based on antibody titers determined using VacciCheck. Figure S1: Specific antibody titers for Feline Panleukopenia Virus (FPV), Feline Herpesvirus type 1 (FHV-1), and Feline Calicivirus (FCV) in 740 Italian cats (titers with an asterisk (*) represent threshold values). Figure S2: Specific antibody titers against Feline Panleukopenia Virus (FPV), Feline Herpesvirus type 1 (FeHV-1), and Feline Calicivirus (FCV) of 115 cats vaccinated at least more than 3 years before sampling (Kruskal–Wallis test).
