# Supplementary material for: Prevalence of Serum Antibody Titers against Core Vaccine Antigens in Italian Cats
Source: Life (Basel). 2023 Nov 23;13(12):2249. doi: 10.3390/life13122249 (PMC10744740; doi:10.3390/life13122249)

Table S1 VacciCheck: correspondence between S scale units and antibody titers, sensitivity, and specificity for Feline Panleukopenia Virus (FPV), Feline Herpesvirus type 1 (FeHV-1), and Feline Calicivirus (FCV)

	**FPV (%)**	**FeHV-1 (%)**	**FCV (%)**
S0	<1:20	<1:4	<1:8
S1	1:20	1:4	1:8
S2	1:40	1:8	1:16
**S3 (threshold)**	**1:80**	**1:16**	**1:32**
S4	1:160	1:32	1:64
S5	1:320	1:64	1:128
S6	1:640	1:128	1:256
>S6	>1:640	>1:128	>1:256
Sensitivity (%)	*98*	*96*	*91*
Specificity (%)	*89*	*93*	*90*

Table S2 Classification of protection categories for Panleukopenia Virus (FPV), Feline Herpesvirus type 1 (FeHV-1), and Feline Calicivirus (FCV) in the 740 Italian cats based on antibody titers of VacciCheck

**Categories**	**FPV**	**FeHV-1**	**FCV**
Threshold values	*1:80*	*1:16*	*1:32*
Unprotected	≤1:20	≤1:4	≤1:8
Weak Positive	>1:20 to <1:80	>1:4 to <1:16	>1:8 to <1:32
Medium Positive	≥1:80 to ≤1:160	≥1:16 to ≤1:32	≥1:32 to ≤1:64
High Positive	>1:160	>1:32	>1:64

Figure S1 Specific antibody titers for Feline Panleukopenia Virus (FPV), Feline Herpesvirus type 1 (FeHV-1), and Feline Calicivirus (FCV) in the 740 Italian cats (titers with asterisk (*) represent the threshold values)

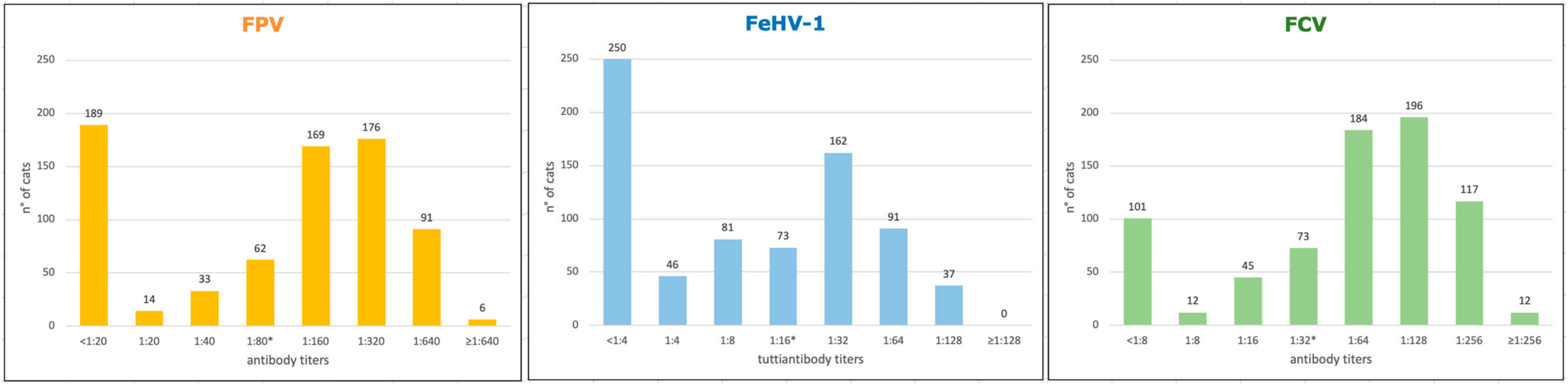

Figure S2 Specific antibody titers against Feline Panleukopenia Virus (FPV), Feline Herpesvirus type 1 (FeHV-1), and Feline Calicivirus (FCV) of the 115 cats vaccinated at least more than 3 years before sampling (Kruskal-Wallis test)

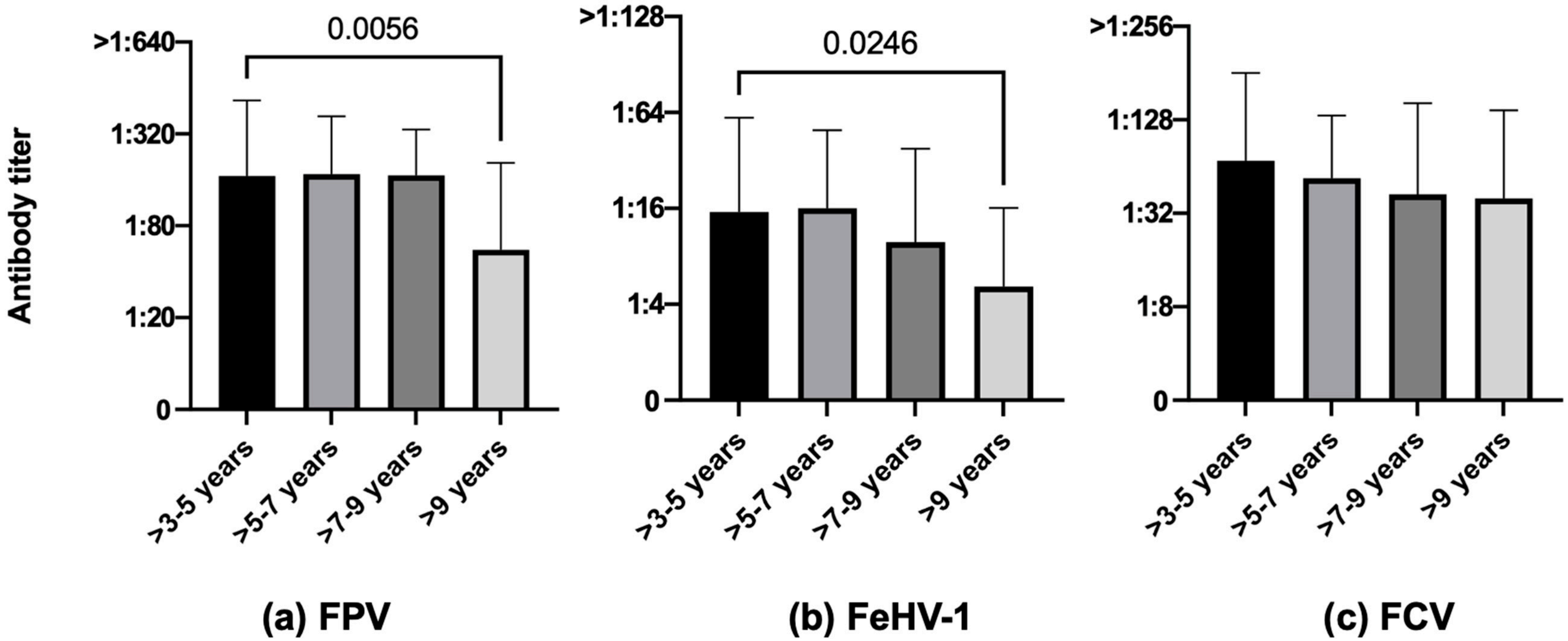

Supplement: Supplementary file 1 [file life-13-02249-s001.zip › life-2619781-supplementary.pdf]
